# Scientific production and impact of citations by scholarship researchers in the area of ophthalmology of CNPq

**DOI:** 10.5935/0004-2749.2024-0413

**Published:** 2025-06-02

**Authors:** Gustavo Dias Macedo, Árlen Almeida Duarte de Sousa, Janini Tatiane Souza Maia, Daniella Reis Barbosa Martelli, Luciano Sólia Násser, Hercílio Martelli Júnior

**Affiliations:** 1 Centro de Pesquisas, Faculdades Unidas do Norte de Minas, Montes Claros, MG, Brazil; 2 Universidade Estadual de Montes Claros, Montes Claros, MG, Brazil

Dear editor,

We read with interest the article entitled, “*Who should finance science? A consideration about publication costs*”^(1)^. The article raises important questions about research funding and scientific publication in Brazil. Over the past two decades, Brazil has experienced significant growth in scientific productivity and international visibility. However, sustaining this growth requires continued financial investments^(2)^.

In Brazil, the National Council for Scientific and Technological Development (CNPq) allocates research funds based on peer evaluation of the merits of the proponent and their proposals. The scientific productivity fellowship (PQ) program is one of the key funding mechanisms of CNPq. PQ fellowships are classified into six levels: A, B, C, D, E, and Senior (https://www.gov.br/cnpq/pt-br/acesso-a-informacao/bolsas-e-auxilios). Previous studies have emphasized the relevance of PQ in various fields of Medicine^(3)^. However, there is a lack of bibliometric studies examining the impact of this funding on Brazilian researchers, particularly in the field of Ophthalmology^(3^,^4)^.

To address this gap, we evaluated technical-scientific indicators, including the H-index, number of articles published, and number of citations in the Web of Science database over the last decade, for PQ-funded researchers in Ophthalmology and researchers working in postgraduate programs in Ophthalmology in Brazil^(4^,^5)^.

A database of 556 Brazilian researchers registered as PQ recipients in Medicine was assessed between May and August 2024 (http://www.bi.cnpq.br/painel/mapa--fomento-cti/). We identified 15 researchers (2.69%) whose main area of research was Ophthalmology. The following 4 dimensions were analyzed: a) researcher profile; b) scientific publications (total career articles published in Journal Citation Reports-indexed journals and total articles published in the last decade, 2014-2023); c) citations received in the Web of Science database (2014-23); and d) H-index.

A database of 63 faculty researchers in postgraduate ophthalmology programs was compiled in February 2025 (https://sucupira.capes.gov.br/). For non-PQ researchers, we analyzed three dimensions: a) number of articles published in the last decade (2014-2023); b) citations received in the Web of Science in the last decade, and c) H-index. Among the 272 postgraduate programs in Medicine, six focused primarily on ophthalmology, all located in the Southeast region: five in the state of São Paulo and one in Minas Gerais.

The Statistical Package for Social Sciences (SPSS), version 27.0 for Windows^®^, was employed for statistical analyses comparing PQ researchers and non-PQ faculty researchers. Data normality was assessed using the Shapiro-Wilk and Kolmogorov-Smirnov tests, with pvalues >0.05 indicating a normal distribution. The Mann-Whitney test was used to evaluate significant differences between the researcher groups.

Among PQ researchers, males predominated (80%), with the majority at level E (46.7%). The 15 Ophthalmology PQ researchers were distributed across four institutions: University of São Paulo, Ribeirão Preto e São Paulo, (n=6; 40%), Federal University of São Paulo (n=7; 46.7%), São Paulo State University (UNESP) (n=1; 6.7%), and Federal University of Minas Gerais (n=1; 6.7%). These PQ researchers published 3,218 scientific articles during their careers (mean publications per researcher=214.53), with 2,908 articles appearing in Journal Citation Reports-indexed journals in the Web of Science database. During 2014-2023, they published 1,264 papers and received 66,092 citations in the Web of Science database. The H-index averaged 28.26, indicating a high level of research productivity among these PQ researchers in Ophthalmology.

Although Ophthalmology has a relatively small number of PQ researchers compared to the top 10 medical specialties (Table 1), it demonstrated high scientific productivity, with a high average number of career publications and a high H-index. Furthermore, when comparing non-PQ researchers to PQ researchers, the latter showed a significantly greater academic impact in the last decade, with higher averages in publications, Web of Science citations, and H-index (Table 2).

**Table 1:** Top ten medical specialties with the highest number of CNPq Research productivity fellows (PQ), (n=556), 2025

Knowledge Area^*^	N
Psychiatry/Neuroscience	50
Endocrinology	48
Gynecology/Obstetrics	48
Cardiology	45
Nephrology/Urology	41
Infectious Diseases/Infectology/Tropical Medicine	41
Hematology and Oncology	34
Neurology	26
Pneumology	24
Genetics/Molecular Biology and Cell	24

* http://www.bi.cnpq.br/painel/mapa-fomento-cti/

**Table 2:** Comparative statistical analysis of researchers with CNPQ research productivity fellowships (PQ) and non-PQ faculty researchers in graduate programs

	Mean	SD	95% CI	Total	p-value^*^
**H-Index**					0.001
With PQ fellowship	28.26	12.69	22.47-35.06	-	
No PQ fellowship	16.44	9.63	14.10-18.83	-	
**Published articles (2014-2023)**					0.000
With PQ fellowship	84.27	39.31	66.21-104.26	1264	
No PQ fellowship	41.83	28.52	35.03-49.27	2635	
**Citations in WoS (2014-2023)**					0.003
With PQ fellowship	4406.13	12566.56	729.05-11152.45	66092	
No PQ fellowship	562.54	794.56	383.44-779.55	35440	

* Mann-Whitney test; - not applicable; WoS= Web of Science.

In conclusion, our study revealed that Brazilian Ophthalmology researchers have a significant academic impact, marked by an increase in citations and published articles over the last decade, as well as an elevated H-index. However, this productivity was mainly concentrated in the Southeast region of the country.
